# Refractory minimal change disease associated with Mycobacterium marinum infection

**DOI:** 10.1007/s13730-026-01131-4

**Published:** 2026-05-12

**Authors:** Yuto Matsui, Kan Katayama, Yuka Mitamura, Keiichi Nagata, Koyo Ohara, Toru Hibi, Yuhei Ito, Naohiro Kawamura, Mayuko Yamakawa, Yuri Oue, Mutsuki Mori, Masahiro Yamawaki, Ryosuke Saiki, Tomohiro Murata, Kaoru Dohi

**Affiliations:** 1 Department of Cardiology and Nephrology, Mie University Graduate School of Medicine, 2-174 Edobashi, Tsu, Mie 514-8507 Japan; 2 Department of Clinical Laboratory, Mie University Hospital, Tsu, Japan; 3 Department of Orthopaedic Surgery, Mie University Graduate School of Medicine, Tsu, Japan; 4 Department of General Medicine, Mie University Hospital, Tsu, Japan; 5 Centre for Rheumatic Diseases, Mie University Hospital, Tsu, Japan

**Keywords:** Minimal change disease, Mycobacterium marinum, Nontuberculous mycobacterial infection, Refractory

## Abstract

This case report describes a rare instance of minimal change disease (MCD) associated with Mycobacterium marinum infection. MCD is typically idiopathic but can occasionally occur secondary to infections. The patient was a 61-year-old fisherman with a history of relapsing MCD who developed a subcutaneous abscess in the lower leg while undergoing rituximab and glucocorticoid therapy. Ziehl-Neelsen staining revealed acid-fast bacilli, and culture at 30 °C isolated M. marinum, a slow-growing nontuberculous mycobacterium (NTM) commonly found in aquatic environments. The infection was attributed to the combination of occupational exposure and immunosuppression. Targeted antimicrobial therapy with clarithromycin, minocycline, and rifampicin led to resolution of the abscess and complete remission of MCD, suggesting a causal link between the infection and disease relapse. This case highlights several key clinical considerations. First, secondary infectious causes should be evaluated in patients with treatment-resistant nephrotic syndrome. Second, appropriate laboratory techniques, including optimized culture conditions, are critical for identifying rare NTM. Third, a detailed assessment of lifestyle and occupational exposures can provide essential diagnostic clues. The report underscores the importance of vigilance in atypical or refractory MCD cases and supports the role of targeted antimicrobial therapy in resolving both infection and associated nephrotic syndrome.

## Introduction

Minimal change disease (MCD) is most commonly idiopathic, but it can also occur secondary to other conditions. Secondary MCD due to infection is rare [1]. Among infectious causes, nephrotic syndrome resulting from nontuberculous mycobacteria (NTM) is exceptionally uncommon, with only a few reported cases, including those involving MCD [2]. We report a rare case of MCD associated with *M. marinum* infection.

*M. marinum* is a slow-growing NTM that typically infects fish, amphibians, and shellfish in saltwater, brackish, and freshwater environments worldwide [3]. Human infection usually occurs through exposure of injured skin to contaminated water, aquatic organisms, or related materials [3]. It is often seen in individuals involved in aquatic activities—such as fishermen, aquarists, oyster farmers, and water sports enthusiasts [4].

## Case report

A 61-year-old man was diagnosed with minimal change disease (MCD) at the age of 44.

He was initially treated with prednisolone and achieved complete remission. However, he experienced a relapse while tapered to prednisolone 2.5 mg. He subsequently achieved complete remission twice with prednisolone 15 mg. The third and fourth relapses were treated with prednisolone 10 mg. While the fifth relapse was also treated with prednisolone 10 mg, the sixth, seventh, and eighth relapses were treated with prednisolone 15 mg and responded well. Because of frequent relapses, mizoribine 100 mg was added to prednisolone 10 mg at the ninth relapse. From the tenth relapse, rituximab 500 mg was administered, and both prednisolone and mizoribine were discontinued after six months. The patient then remained relapse-free for four years. However, he subsequently developed recurrent nephrotic syndrome. Although partial response to prednisolone 30 mg was observed, complete remission was not achieved, and he received rituximab (500 mg/body) again at age 57, followed by two additional doses at six-month intervals starting at age 60, resulting in complete remission. His B-cell levels were sufficiently suppressed. His medical history included hypertension and hyperuricemia, both managed with sacubitril valsartan sodium hydrate 100 mg and febuxostat 20 mg, respectively. At age 50, he underwent resection of sigmoid colon polyps. He was employed as a fisherman.

At presentation, he developed bilateral leg edema and experienced a weight gain of 9 kg. Laboratory findings showed severe hypoalbuminemia with a serum albumin level of 1.1 g/dL and proteinuria of 15.2 g/g creatinine (Table 1). A relapse of MCD was diagnosed.

**Table 1:** Laboratory data

Urinary examination		Alb (g/dl, 4.1–5.1)	1.1
pH (4.5–7.5)	6	BUN (mg/dl, 8–20)	46.8
Protein (g/gCr)	15.21	Cr (mg/dl, 0.46–0.79)	1.84
Occult blood	(2+)	eGFR (ml/min/1.73m^2^)	30.6
RBC (/ high power field)	< 1	UA (mg/dl, 2.6–5.5)	6.8
Fatty casts (/whole field)	100–999	Na (mEq/l, 138–145)	141
		K (mEq/l, 3.6–4.8)	5.1
Complete blood count		Cl (mEq/l, 101–108)	117
WBC (/µl, 3300–8600)	6640	Ca (mg/dl, 8.8–10.1)	8.0
RBC (×10^4^/µl, 435–555)	534	IP (mg/dl, 2.7–4.6)	4.3
Hb (g/dl, 13.7–16.8)	15.6	AST (U/l, 13–30)	19
Plt (×10^4^/µl, 15.8–34.8)	35.6	ALT (U/l, 7–23)	15
		LDH (U/l, 124–222)	321
Blood chemistry		ALP (U/l, 38–113)	97
HbA1c (%, 4.9-6.0)	6	γGTP (U/l, 9–32)	18
Glu (mg/dl, 73–109)	101	CRP (mg/dl, 0-0.14)	0.2
TP (g/dl, 6.6–8.1)	3.7	T-Chol (mg/dL, 142–248)	507

Alb, albumin; ALP, alkaline phosphatase; ALT, alanine transaminase; AST, asparate transaminase; BUN, blood urea nitrogen; Ca, calcium; Cl, chloride; Cr, creatinine; CRP, C-reactive protein; eGFR, estimated glomerular filtration rate; Glu, glucose; γGTP, γ-glutamyltranspeptidase; Hb, hemoglobin; HbA1c, hemoglobin A1c; IP inorganic phosphate; K, kalium; LDH, lactate dehydrogenase; Na, natrium; Plt, platelets; RBC, red blood cells; TP, total protein; T-Chol, total cholesterol; UA, uric acid; WBC, white blood cells

On physical examination, his height was 167 cm, weight 74.5 kg, and body mass index 27.1. His body temperature was 35.8 °C, blood pressure 162/90 mmHg, oxygen saturation 97% on room air, and pulse 73 beats per minute. Cardiac examination revealed no murmurs, and lung auscultation was clear.

For joint pain, he had been taking celecoxib, which was switched to acetaminophen to avoid potential worsening of kidney function.

**Fig. 1:**
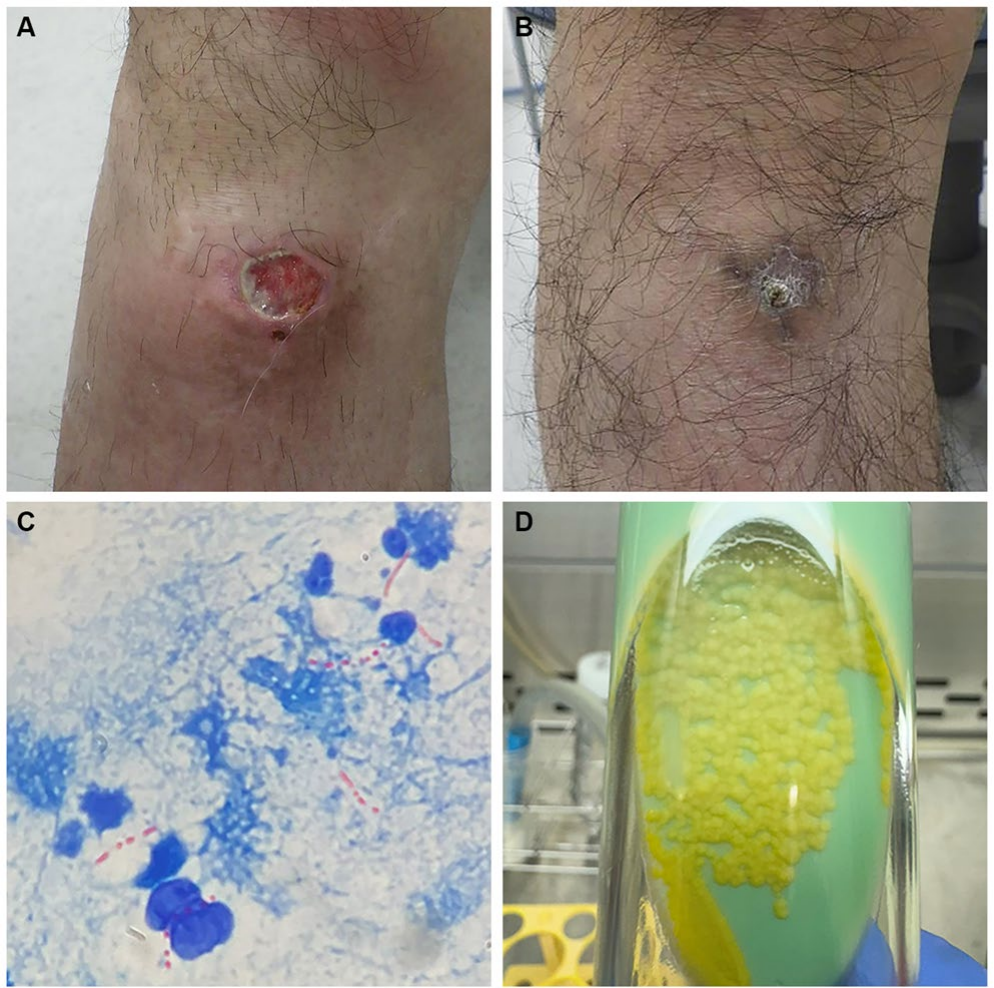
Diagonosis of Mycobacterium marinum. **A** Ulcer formation in the right lower leg. **B** Ulcer formation was cured after antibiotics treatment. **C** Ziehl-Neelsen staining. **D** Growth was observed on Ogawa medium at 30 °C

To treat the relapse of nephrotic syndrome, he received rituximab (500 mg/body), along with oral prednisolone and furosemide for three months. Although his serum albumin level and edema improved, proteinuria persisted. In the fourth month of treatment, he noticed redness and purulent discharge at the medial malleolus of his right lower leg (Fig. 1A). Computed tomography (CT) revealed a subcutaneous abscess in the medial malleolus, without evidence of gas formation or osteomyelitis.

Aspiration and culture of the abscess were performed. Gram staining did not reveal any organisms, prompting Ziehl-Neelsen staining, which was positive for acid-fast bacilli (Fig. 1C). Given the patient’s occupation as a fisherman, *M. marinum* infection was suspected. Cultures were set up on both mycobacteria growth indicator tube (MGIT) medium at 37 °C and Ogawa medium at 30 °C. After 10 days, growth was observed on Ogawa medium (Fig. 1D), and *M. marinum* was identified by mass spectrometry. The MGIT culture at 37 °C remained negative after 42 days.

**Fig. 2:**
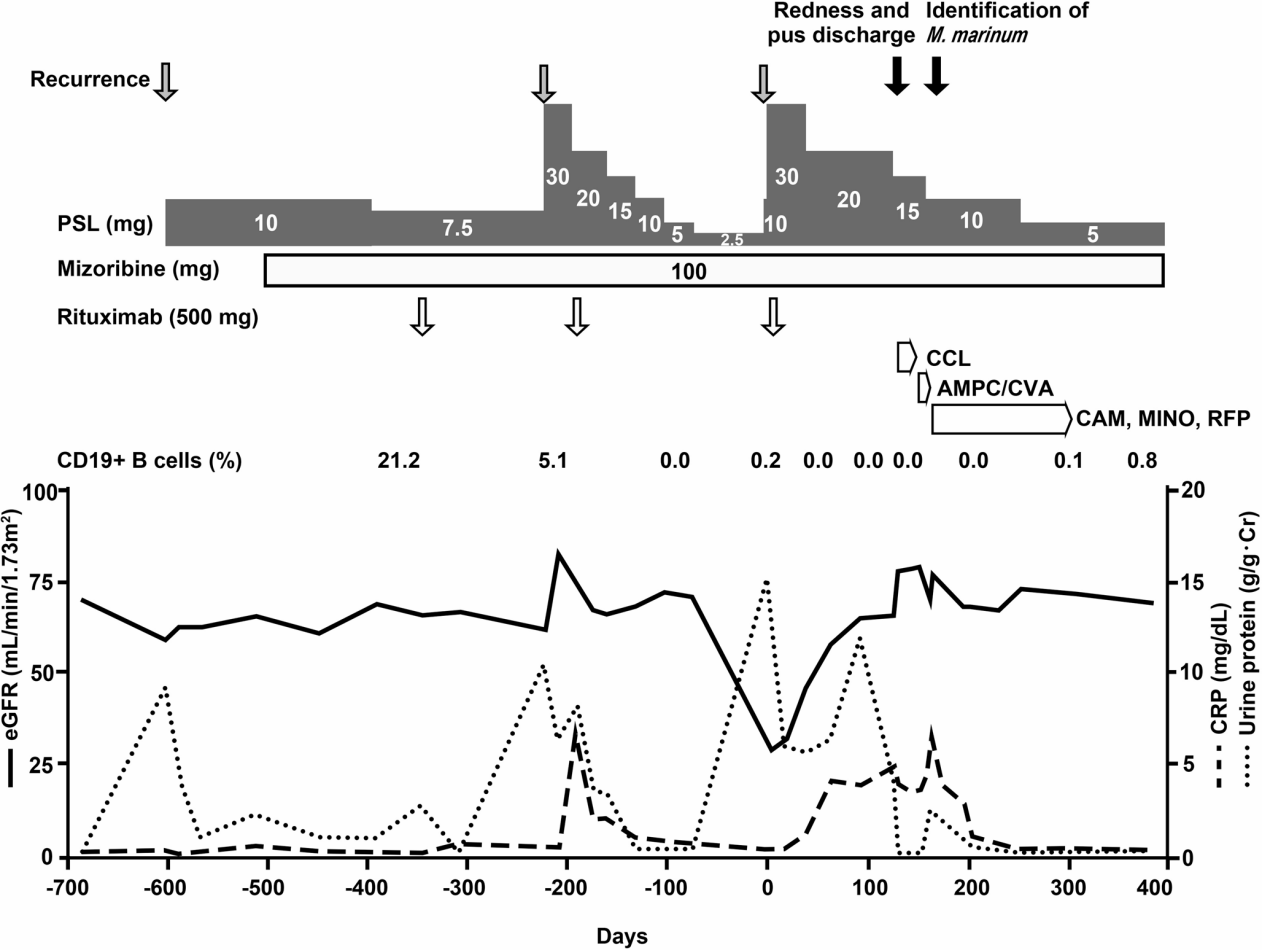
The clinical course. AMPC/CVA, amoxicillin/clavulanic acid; CCL, cefaclor; CAM, clarithromycin; CRP, C-reactive protein; eGFR, estimated glomerular filtration rate; *M. marinum*, Mycobacterium marinum; MINO, minocycline; PSL, prednisolone; RFP, rifampicin

Empiric antibiotic therapy with cefaclor was continued for 21 days, followed by amoxicillin/clavulanic acid for 11 days until susceptibility results were available (Fig. 2). Prednisolone was tapered to 10 mg and maintained. Thirty-two days after sample collection, drug susceptibility testing confirmed sensitivity to clarithromycin, treatments with clarithromycin, minocycline, and rifampicin were subsequently administered. After two months of targeted antibiotic therapy, the abscess resolved (Fig. 1B), white blood cell count normalized, and the patient achieved complete remission of proteinuria.

## Discussion

*M. marinum* infection in humans is uncommon, with an annual incidence of 0.27 per 100,000 people in the United States and 0.04 per 100,000 in France [4, 5]. The organism grows optimally on Lowenstein-Jensen medium at 30 °C, requiring 2–3 weeks for visible growth, although cultures should be maintained for at least 6 weeks to confirm diagnosis [3, 4]. Importantly, *M. marinum* does not grow at 37 °C, the standard incubation temperature in most laboratories [3, 4]. In our case, the possibility of mycobacterial infection was first raised after acid-fast bacilli were identified on Ziehl-Neelsen staining. This prompted a thorough re-evaluation of the patient’s occupational and lifestyle history, revealing that he was a fisherman and immunosuppressed—factors that significantly increased his risk for *M. marinum* infection. This insight allowed us to adjust culture conditions appropriately and confirm the diagnosis.

According to Bhatty et al., *M. marinum* infections can be classified into four types:

Type 1: Superficial skin infection in immunocompetent individuals.

Type 2: Single or multiple abscesses and subcutaneous granulomas, with or without ulceration in immunosuppressed individuals.

Type 3: Deep infections such as tenosynovitis, synovitis, or osteomyelitis.

Type 4: Disseminated infections in immunocompromised hosts, often involving pulmonary or systemic manifestations [3, 6].

Our case was consistent with a type 2 infection. Deep infections caused by *M. marinum* may require surgical intervention and are best assessed using magnetic resonance imaging [7]. In our patient, CT helped localize the infection site, and surgical drainage was performed accordingly.

In terms of antimicrobial therapy, *M. marinum* is inherently resistant to several drugs, including streptomycin, azithromycin, isoniazid, and pyrazinamide. It also shows high minimum inhibitory concentrations for levofloxacin, ciprofloxacin, and older quinolones [3–5]. Effective regimens for deep infections generally involve two or more agents, such as rifampicin, moxifloxacin, ethambutol, clarithromycin, linezolid, and tetracycline. In this case, we opted for a three-drug combination therapy.

While most MCD cases are primary, secondary causes include malignancies, drug reactions, autoimmune diseases, and infections [1]. Secondary MCD due to infection is particularly rare, but has been associated with syphilis, tuberculosis, human immunodeficiency virus, mycoplasma, ehrlichiosis, Echinococcus, and Schistosoma infections. In many of these cases, treating the underlying infection led to improvement or resolution of the nephrotic syndrome [1]. Regarding infection-associated MCD, remission has been reported to be achieved at a median of 20 months after rituximab administration in HIV-associated MCD, and this has been suggested to be associated not with HIV infection itself, but rather with CMIP induction [8].

There have been reports of kidney diseases associated with NTM infections, including membranous nephropathy linked to Mycobacterium shimoidei [9], crescentic glomerulonephritis with Mycobacterium avium [10], and infection-related nephritis due to Mycobacterium leprae [11]. ANCA-associated vasculitis with NTM positivity was not treatment-resistant [12]. One report even describes MCD associated with pulmonary M. avium disease, which improved with antimycobacterial therapy [2]. However, to our knowledge, this is the first reported case of MCD associated with *M. marinum* infection.

Our case also resembles a report by Jones et al., in which an 8-year-old boy with frequently relapsing MCD became refractory to treatment until a concurrent tuberculous osteitis was diagnosed and treated, resulting in complete and sustained remission [13]. Similarly, in our patient, frequently relapsing MCD that responded well to glucocorticoids and rituximab became refractory to treatment and achieved remission after treatment for M. marinum infection. *At day − 600*,* we did not consider the patient to be treatment-resistant. At days − 350 and − 200*,* rituximab administration was followed by a favorable reduction in proteinuria*,* and we considered the patient to be responsive to treatment at those time points. However*,* after rituximab administration at day 0*,* the subsequent improvement in proteinuria was clearly less pronounced*,* leading us to consider the disease as treatment-resistant at that stage.* We thought that treatment for the infection improved the response to glucocorticoids to the same level as last time. Although we did not perform a second kidney biopsy, and therefore cannot rule out the possibility of another condition, the temporal association between infection treatment and remission strongly suggests a causal relationship. This highlights the importance of considering secondary causes—especially infections—in cases of treatment-resistant nephrotic syndrome, even in patients with a history of frequent relapses responsive to glucocorticoids.

The limitation of the present case is that anti-nephrin antibodies were not measured, despite the possibility that the cells producing anti-nephrin antibodies—previously suppressed by rituximab—may have shifted from CD20-positive plasma cells to CD38-positive plasma cells.

In conclusion, we report a case of MCD associated with *M. marinum* infection. Accurate identification of NTM requires tailored diagnostic approaches, including extended incubation at appropriate temperatures. A detailed occupational and lifestyle history is essential for guiding clinical suspicion and ensuring proper microbial culture conditions.
